# Early Mortality Among Peritoneal Dialysis and Hemodialysis Patients Who Transitioned With an Optimal Outpatient Start

**DOI:** 10.1016/j.ekir.2018.10.008

**Published:** 2018-10-16

**Authors:** Hui Zhou, John J. Sim, Simran K. Bhandari, Sally F. Shaw, Jiaxiao Shi, Scott A. Rasgon, Csaba P. Kovesdy, Kamyar Kalantar-Zadeh, Michael H. Kanter, Steven J. Jacobsen

**Affiliations:** 1 Department of Research and Evaluation, Kaiser Permanente Southern California, Pasadena, California, USA; 2 Division of Nephrology and Hypertension, Kaiser Permanente Los Angeles Medical Center, Los Angeles, California, USA; 3 Division of Nephrology, University of Tennessee Health Science Center, Memphis, Tennessee, USA; 4 Division of Nephrology and Hypertension, University of California Irvine Medical Center, Irvine, California, USA; 5 Regional Quality and Clinical Analysis, Southern California Permanente Medical Group, Kaiser Permanente Southern California, Pasadena, California, USA

**Keywords:** hemodialysis, mortality, optimal end-stage renal disease transition, peritoneal dialysis, propensity-matched cohort

## Abstract

**Introduction:**

Lower early mortality observed in peritoneal dialysis (PD) compared with hemodialysis (HD) may be due to differential pre–end-stage renal disease (ESRD) care and the stable setting of transition to dialysis where PD starts are more frequently outpatient rather than during an unscheduled hospitalization. To account for these circumstances, we compared early mortality among a matched cohort of PD and HD patients who had optimal and outpatient starts.

**Methods:**

Retrospective cohort study performed among patients with chronic kidney disease (CKD) who transitioned to ESRD from 1 January 2002 to 31 March 2015 with an optimal start in an outpatient setting. Optimal start defined as (i) HD with an arteriovenous graft or fistula or (ii) PD. Propensity score modeling factoring age, race, sex, comorbidities, estimated glomerular filtration rate (eGFR) level, and change in eGFR before ESRD was used to create a matched cohort of HD and PD. All-cause mortality was compared at 6 months, 1 year, and 2 years posttransition to ESRD.

**Results:**

Among 2094 patients (1398 HD and 696 PD) who had optimal outpatient transition to ESRD, 541 HD patients were propensity score–matched to 541 PD patients (caliper distance <0.001). All-cause mortality odds ratios (OR) in PD compared with HD were 0.79 (0.39–1.63), 0.73 (0.43–1.23), and 0.88 (0.62–1.26) for 6 months, 1 year, and 2 years, respectively. Time-varying analysis accounting for modality switch (19% PD, 1.9% HD) demonstrated a mortality hazard ratio of 0.94 (0.70–1.24)

**Conclusion:**

Among an optimal start CKD cohort that transitioned to ESRD on an outpatient basis, we found no evidence of differences in early mortality between PD and HD.

## Graphical abstract

**Figure:**
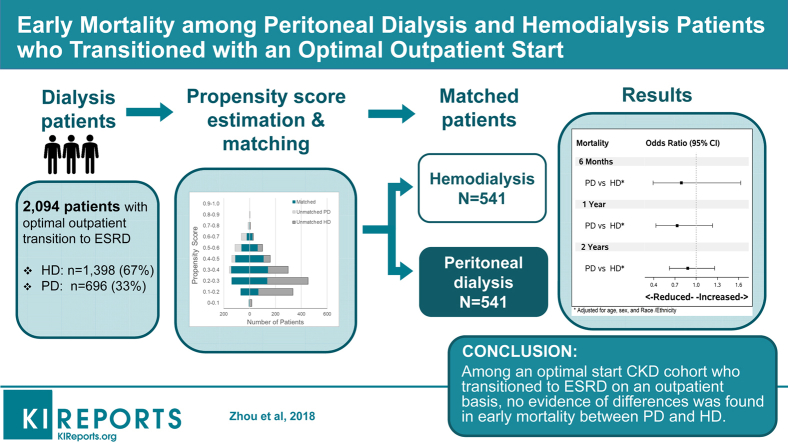

Ideal management strategies before and at the initiation of dialysis to optimize the transition to ESRD and improve outcomes have not been well defined.^1^ Improving our understanding of this transition period has important implications, as ESRD burdens more than 600,000 people in the United States with a 5-year survival of approximately 50%.^2^ Although overall mortality rates of the ESRD population are improving, the immediate period after transition to ESRD is associated with the highest mortality rates. Mortality rates up to 30% have been described within the first year of transition from CKD to ESRD.^3^, ^4^, ^5^, ^6^ Improved understanding of patient needs and circumstances at the transition and early ESRD stages may ultimately help improve survival and direct pre-ESRD treatment strategies.^1^

An important consideration is whether a survival advantage exists between HD and PD modalities. Demonstrating a mortality benefit would affect dialysis modality choice and clinical management strategies before ESRD. Dialysis modality selection is generally based on a multitude of factors, including patient and physician preference, comorbidities, logistical consideration of location of treatment facilities, and acuity/timing of uremia. Several observational studies have attempted to compare survival among PD and HD patients, but overall the findings have been inconsistent. Some observations have shown an initial survival advantage among PD patients in the first 2 years after ESRD transition, after which mortality risk in PD increased; whereas other studies have shown no clear mortality benefit between PD and HD with the differences in mortality risk being attributed to possible residual confounding.^7^, ^8^, ^9^, ^10^ Overall, the interpretation of past comparisons evaluating the differences between HD and PD have been limited due to selection and indication bias.^11^ A comparison restricted to individuals who are eligible for both modalities would provide additional insights on survival benefit.^12^

We hypothesize that lower early mortality observed in PD compared with HD may be due to differential pre-ESRD care and the stable transition to dialysis where PD starts are more frequently outpatient rather than during a hospitalization. We previously reported on a large diverse CKD cohort that transitioned to ESRD where mortality rates were highest within 1 month after ESRD.^13^ HD patients had an increased mortality risk at 6 months compared with PD; however, we observed that nearly all PD patients (97%) transitioned to ESRD in an outpatient setting. Thus, inpatient starts and nonoptimal HD starts may have confounded the comparisons between the PD and HD early mortality. To account for these circumstance, we sought to compare 6-month, 1-year, and 2-year mortality among PD and HD patients who had optimal, outpatient dialysis initiation among a large diverse population for a routine clinical practice environment. Given the confounding by indication bias, the effect of treatment cannot be appropriately evaluated by directly comparing outcomes between treatment groups in an observational study. Application of propensity score methods to account for the probability of treatment selection based on observed baseline covariates has helped in determining more unbiased estimates using observational data.^14^, ^15^ Therefore, we performed a comparison of survival outcomes among a propensity score–matched cohort of PD and HD patients.

## Methods

### Study Population

A retrospective cohort study of Kaiser Permanente Southern California (KPSC) members identified between January 1, 2002, and March 31, 2015, was performed. KPSC is a prepaid integrated health system composed of 14 medical centers and more than 200 satellite clinics that provides comprehensive care to more than 4.2 million members throughout Southern California. As of December 31, 2017, there were more than 2.5 million adult members within KPSC. The membership and CKD patient population is racially, ethnically, and socioeconomically diverse, reflecting the general population of Southern California.^16^, ^17^, ^18^ All KPSC members have similar benefits and access to health care services, clinic visits, procedures, and copays for medications. Complete health care encounters are tracked using a common electronic health record from which all study information was extracted. All data of this study were collected as part of routine clinical encounters in which health care providers determined the need for laboratory measurements, procedures, and medications. The study protocol was reviewed and approved by the KPSC Institutional Review Board (#10591) and was exempt from obtaining informed consent.

The study population has been previously described.^13^ In brief, the study population included patients with CKD aged 18 years and older who transitioned to ESRD and had at least 1 serum creatinine measurement within 90 days before ESRD. eGFR was estimated from serum creatinine levels using the Chronic Kidney Disease Epidemiology Collaboration Equation.^19^ CKD was defined as a minimum of 2 outpatient creatinine measurements 90 days or more apart that demonstrated an eGFR <45 ml/min per 1.73 m^2^. All patients were required to have a minimum of 6 months of continuous membership in the health plan before ESRD transition to reliably capture comorbidities. The date of transition to ESRD was used as the index date.

### Definitions of Optimal Start ESRD

ESRD was defined as treatment with HD (in center, home, or nocturnal) or PD. All patients were required to initiate dialysis (HD or PD) on an outpatient basis with an optimal start. Optimal start was defined as (i) initiation of HD with an arteriovenous fistula or arteriovenous graft, or (ii) transition with PD. Patients who initiated dialysis in an inpatient setting, started HD with a central venous catheter, or received a preemptive renal transplant were excluded.

### Data Collection and Laboratory Measurements

All laboratory data, vital sign assessments (including blood pressure measurements), and diagnostic and procedures codes are recorded and collected into the electronic health record and in KPSC regional quality information data sources.^17^ Comorbidities including hypertension, diabetes mellitus (DM), and congestive heart failure (CHF), were assessed based on inpatient and outpatient International Classification of Diseases diagnoses coding. Acute kidney injury (AKI) was also determined by International Classification of Diseases coding. The Deyo adaption of the Charlson comorbidity index was determined using International Classification of Diseases diagnoses codes from inpatient and outpatient encounters as an overall measure of disease burden.^20^ All laboratory measurements were performed from an American College of Pathology/Clinical Laboratory Improvement Act–certified laboratory. When available, laboratory values on serum albumin, hemoglobin, calcium, potassium, bicarbonate, blood urea nitrogen, phosphorus, ferritin, iron, and hemoglobin A1C were extracted. Baseline characteristics, laboratory data, and all comorbidities were evaluated in the 90 days before ESRD transition. In addition, information on type of dialysis access, and AKI coded within 90 days before ESRD were extracted. Data on hospitalizations and diagnoses that occurred outside the health system were extracted through administrative billing and claims records.

### Outcomes

The primary outcome evaluated was all-cause mortality. Mortality information was obtained from the KPSC Mortality Database, which combines information from 7 data sources, including California State Death Master Files, California State Multiple Causes of Death Master Files, Social Security Administration Death Master Files, KPSC Hospital and Emergency Room records, KPSC Membership System, Perinatal Data Mart, and Outside Claims Processing System. In sensitivity analyses, individuals were followed up to 2 years following ESRD transition, until death, disenrollment from the health plan, or until the end of the study period (March 31, 2017).

### Statistical Analyses

The characteristics of patients on PD and HD were compared. Student *t* test or nonparametric Kruskal-Wallis tests were used for comparison of continuous variables as appropriate, and χ^2^ tests were used for comparison of categorical variables. Percent change in eGFR was estimated by the difference between first and last eGFR measured within 90 days before transition was calculated for each patient.

Mortality rates were calculated for each month following ESRD transition up to 2 years and reported as events per 1000 patient-years. The primary analysis was to compare the risk of all-cause mortality among PD versus HD patients. Logistic regression modeling was used to estimate ORs for mortality in patients who transitioned to PD versus HD at 6 months, 1 year, and at 2 years. Multivariable ORs were estimated with adjustment for potential confounders including age, gender, race/ethnicity, Charlson comorbidity index, preexisting DM and CHF, AKI, eGFR before ESRD, baseline bicarbonate, and percent change in eGFR before ESRD.

To minimize potential selection bias and confounding by indication, a propensity score–matched cohort of HD and PD patients was created. In brief, the propensity score was created to represent the probability of being treated by PD as the initial dialysis modality using logistic regression. Baseline variables including age, sex, race/ethnicity, preexisting DM and CHF, AKI, eGFR, bicarbonate, and change percent of eGFR in 90 days before dialysis were used as independent predictors. C-statistics were used to evaluate the model performance. HD patients were matched 1:1 to PD patients with similar propensity scores and a caliper distance ≤ 0.001. Baseline covariates related to treatment selection were compared before and after matching. The ratio of hazards for HD versus PD was not constant in 2 years and thus the proportional hazards assumption was violated. Therefore, we performed multivariable logistic regression modeling adjusting for age, sex, and race/ethnicity to estimate ORs for mortality at 6 months, 1 year, and 2 years, respectively, among the matched PD versus HD patients.

To account for change in dialysis modality in the 2 years after ESRD transition, a sensitivity analysis was conducted. Time-varying survival analysis was performed to estimate hazard ratios for all-cause mortality between PD and HD with adjustment for age, sex, race/ethnicity, DM, CHF, AKI, eGFR, bicarbonate, and the percent change in eGFR in the 90 days before dialysis.

All statistical analyses were generated using the SAS Enterprise Guide (version 5.1; SAS Institute, Cary, NC). Results with *P* < 0.05 were considered statistically significant.

## Results

### Cohort Characteristics

**Figure 1:**
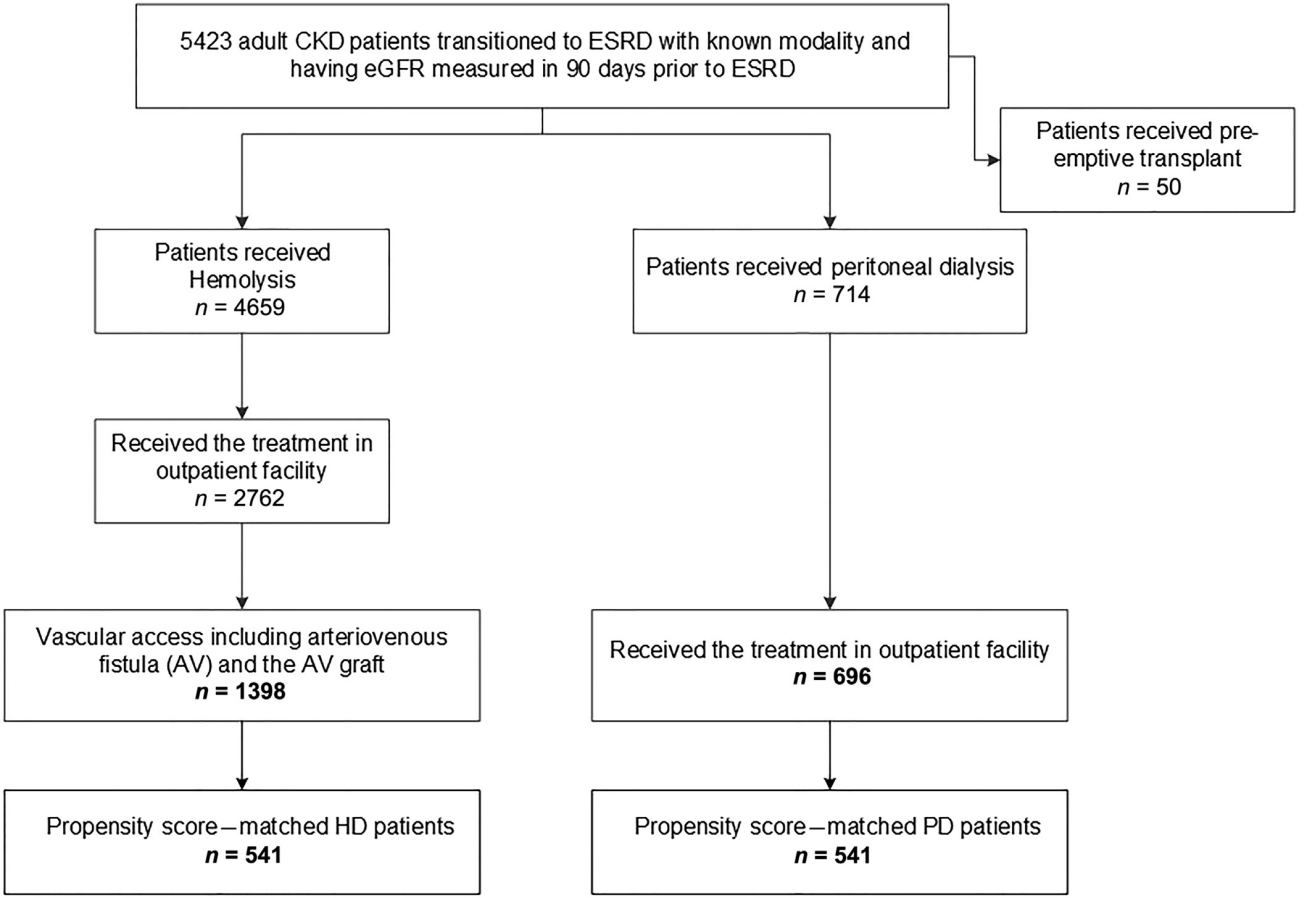
Study population. Among 5423 patients with chronic kidney disease (CKD) with 6-month continuous membership, information on pre–end-stage renal disease (ESRD) estimated glomerular filtration rate (eGFR), and identified dialysis modality, 2094 patients transitioned to ESRD with an optimal start and in an outpatient setting. Propensity score matching, which accounted for age, sex, acute kidney injury within 90 days before ESRD, eGFR before dialysis, diabetes, congestive heart failure, Charlson comorbidity index, and bicarbonate, generated a peritoneal dialysis (PD) (541) and hemodialysis (HD) (541) cohort who were similar in characteristics and likely to receive either modality.

A total of 5423 patients with CKD who had an eGFR measurement 90 days transitioned to ESRD in the observation period. After excluding patients initiating dialysis in the inpatient setting or without an optimal start, 2094 patients were identified for inclusion in the study (Figure 1). Among this outpatient optimal start cohort, 1398 (66.8%) transitioned to HD and 696 (33.2%) transitioned to PD.

The mean age was 62 (SD 12.9) years with 39.9% women. The race/ethnicity composition of the population was 25.9% non-Hispanic white, 20.7% black, 39.0% Hispanic, and 12.0% Asian (Table 1). DM was present in 77.7%, whereas hypertension was diagnosed and coded for nearly the entire study cohort. In the entire study population, 6.4% had an eGFR of ≥15 ml/min per 1.73 m^2^ before ESRD transition.

**Table 1:** Characteristics of patients with CKD who transitioned to HD and PD before and after propensity score matching

Characteristics	All ESRD	Full cohort			After propensity matching		
		HD	PD	*P*	HD	PD	*P*
*n* (%)	2094 (100)	1398 (66.8)	696 (33.2)		541 (50.0)	541 (50.0)	
Age, mean (SD)	61.9 (12.9)	64.2 (12.1)	57.1 (13.3)	<0.001	61.1 (12.4)	60.0 (12.3)	0.3
Female, %	39.9	39	41.7	0.2	41.6	38.8	0.4
Race, %				0.5			0.4
White	25.9	26.4	25		25.5	26.6	
Black	20.7	21.7	18.8		23.1	18.7	
Hispanic	39.0	38.1	40.7		37.2	39.2	
Other	14.4	13.8	15.6		14.2	15.5	
Diabetes mellitus, %	77.7	80.1	72.8	<0.001	75.6	78.4	0.3
Hypertension, %	99.6	99.6	99.6	0.4	99.6	99.8	0.6
Congestive heart failure, %	55.8	59.7	47.8	<0.001	52.7	54.7	0.5
Charlson comorbidity score				<0.001			0.6
2, %	13	5.1	9.2		7.4	7.6	
3–4, %	20.2	22.5	33.6		28.7	25.9	
≥5, %	66.7	72.5	57.2		64.0	66.5	
Acute kidney injury	16.3	16.9	15.2	0.3	15.5	16.8	0.6
eGFR, ml/min per 1.73 m^2^				0.003			1
<5	4.8	5.6	3.3		4.1	4.1	
5–9	52.7	54.5	49.1		50.6	50.5	
10–14	36.1	33.7	40.8		38.8	39	
≥15	6.4	6.2	6.8		6.5	6.5	
Potassium, mean (SD)	4.5 (0.62)	4.5 (0.62)	4.5 (0.61)	0.1	4.5 (0.61)	4.5 (0.61)	0.1
Ferritin, mean (SD)	311.3 (281.0)	318.9 (282.3)	297.5 (278.2)	0.02	311.5 (268.8)	310.6 (282.7)	0.5

CKD, chronic kidney disease; eGFR, estimated glomerular filtration rate; ESRD, end-stage renal disease; HD, hemodialysis; PD, peritoneal dialysis.

**Table 2:** Hazard ratios for mortality in 2 years among all PD versus HD patients using intention-to-treat analysis and time-varying analysis

Outcome	Intention-to-treat analysis		Time-varying analysis considering switch	
	Crude	Adjusted^a^	Crude	Adjusted^a^
2-year mortality	0.71 (0.54–0.92)	0.95 (0.73–1.25)	0.67 (0.51–0.89)	0.94 (0.70–1.24)

HD, hemodialysis; PD, peritoneal dialysis. Hazard ratios (95% confidence interval) were provided.

Values in parentheses represent 95% confidence intervals.

a Adjusted for age, sex, acute kidney injury within 90 days before end-stage renal disease, estimated glomerular filtration rate before dialysis, diabetes, congestive heart failure, Charlson comorbidity index, and bicarbonate.

### PD Versus HD

Among the 2094 patients, HD patients were older (64 vs. 57 years) and had a greater percentage with Charlson comorbidity index ≥5 (73% vs. 57%). HD patients had higher rates of preexisting DM (80% vs. 73%) and CHF (60% vs. 48%) compared with the PD patients. Propensity score matching resulted in 541 (78%) PD and 541 (39%) HD patients with similar characteristics, which suggested these patients were likely to be eligible for both modalities (Table 1). Comparison between the matched cohort and nonmatched population demonstrated no significant differences in sex, race/ethnicity, preexisting DM, hypertension, and AKI. However, patients in the matched cohort were younger (61 years vs. 63 years) and had a lesser proportion of patients starting dialysis with eGFR <5 ml/min per 1.73 m^2^ (Supplementary Table S1).

### Outcomes

**Figure 2:**
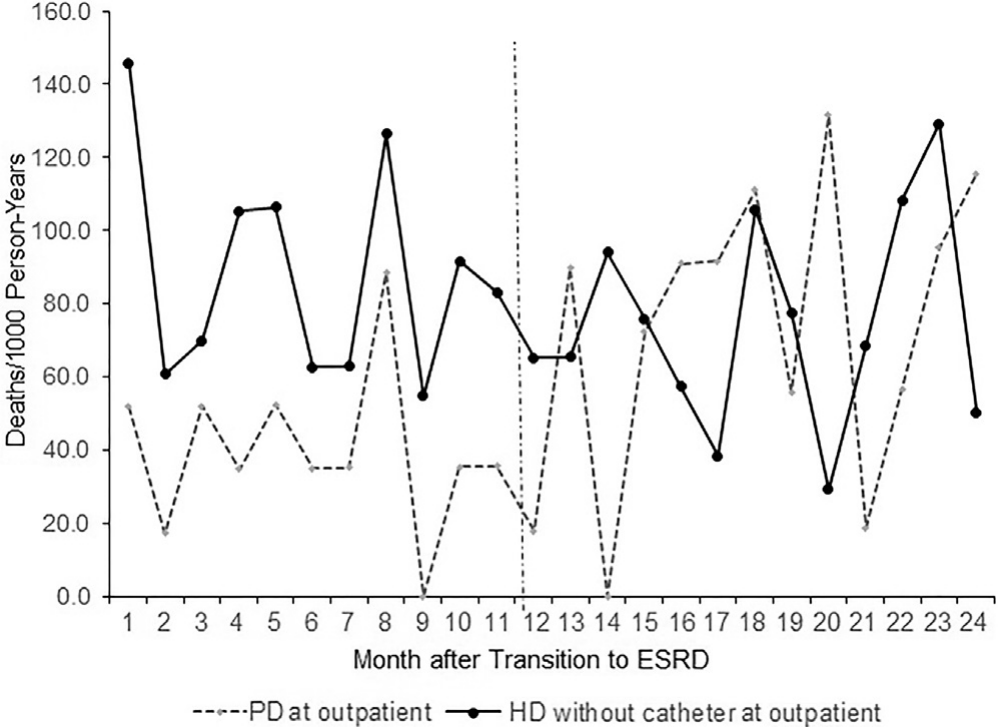
Monthly crude mortality rates (deaths per 1000 patient years) by modality type in 2 years after end-stage renal disease (ESRD) transition among 2094 total unmatched population with HD (1398) and peritoneal dialysis (PD) (696) patients.

**Figure 3:**
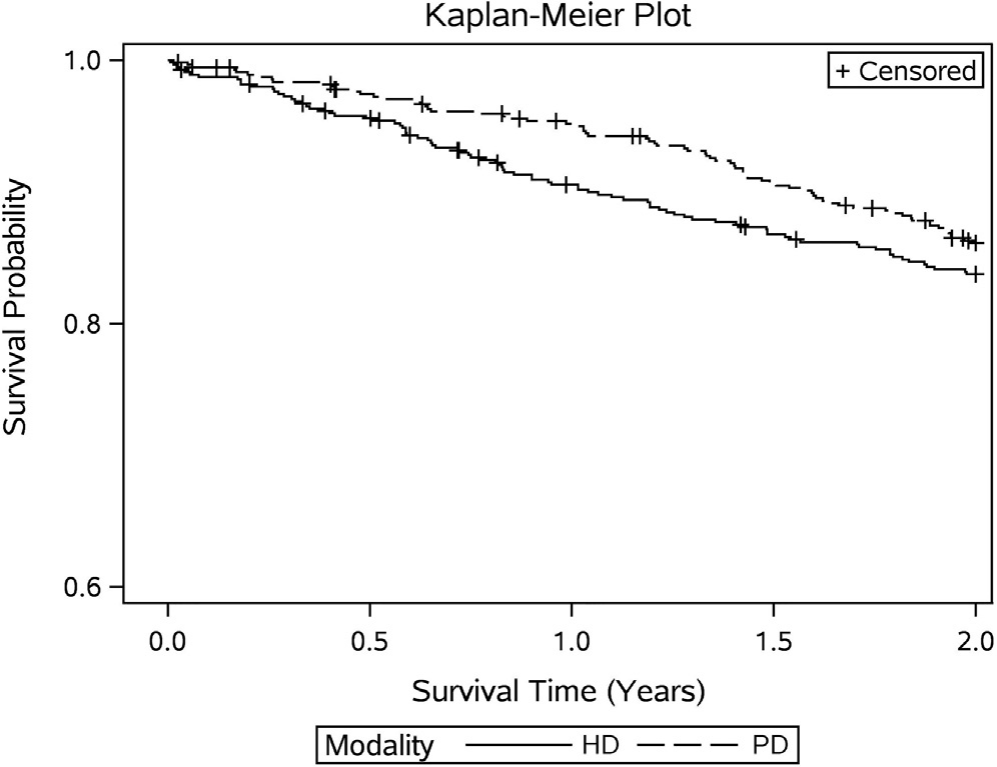
Kaplan-Meier survival curve comparing mortality in the 2 years after end-stage renal disease transition among propensity score–matched peritoneal dialysis (PD) (541) and hemodialysis (HD) (541) patients.

Overall, 286 (13.7%) death events occurred within 2 years after ESRD initiation among the total study cohort; 77 (3.7%) and 142 (6.7%) within 6 months and 1 year, respectively. The crude mortality in the PD cohort was lower than the HD cohort at 6 months post-ESRD transition with 20 versus 45 (deaths per 1000 HD person-years) but not at 1 or 2 years (Figure 2, Supplementary Table S2). Among the propensity score–matched cohorts, no significant differences in survival curve were observed in the 2-year observation window (Figure 3).

### Regressions

**Figure 4:**
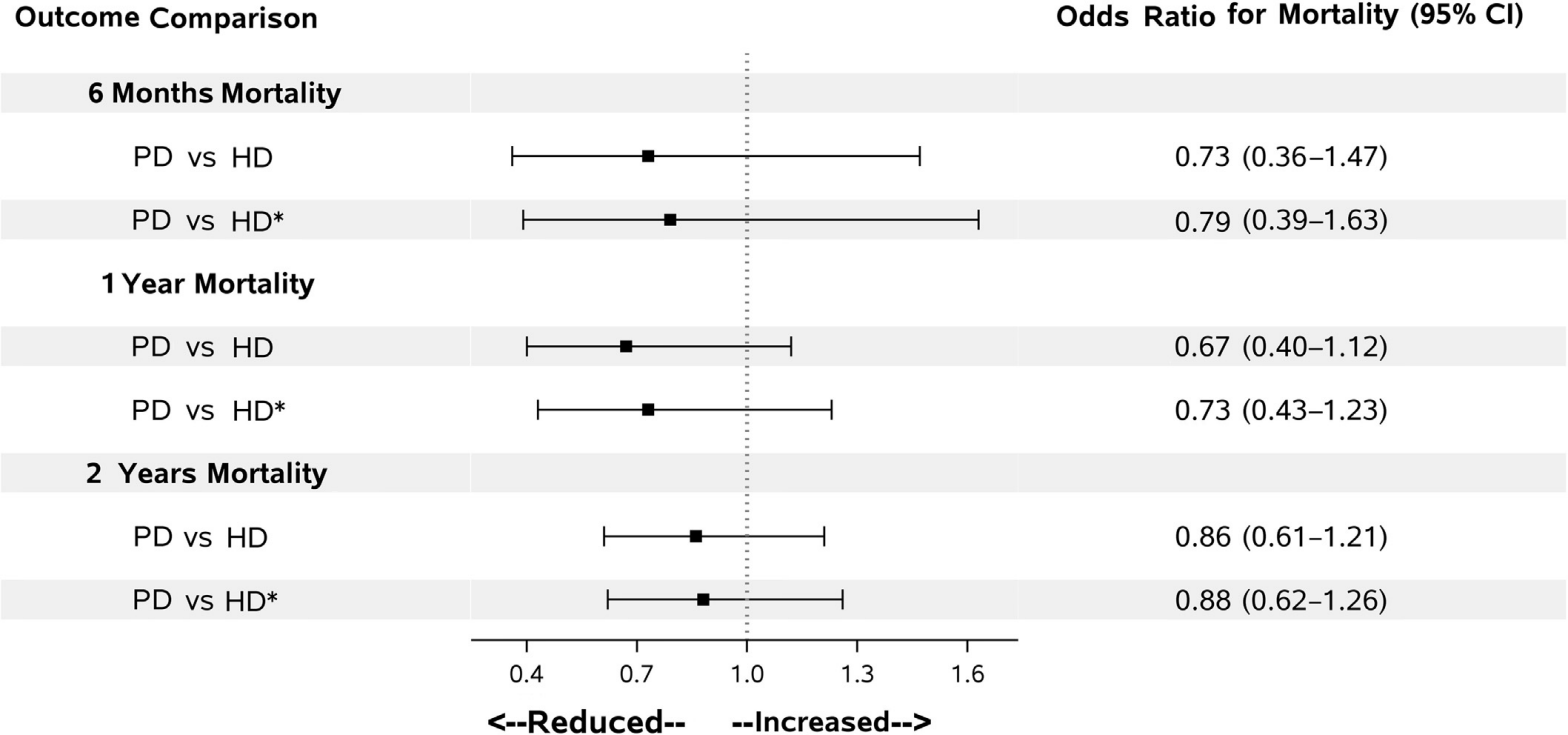
Among the propensity-matched cohorts, crude and adjusted odds ratio of mortality in peritoneal dialysis (PD) versus hemodialysis (HD) at 6 months, 1 year, or 2 years. Adjusted odds ratios accounted for age, sex, and race/ethnicity. CI, confidence interval.

After adjustment for age, gender, and race/ethnicity, the propensity score–matched PD cohort had an all-cause mortality adjusted OR (95% confidence interval) of 0.79 (0.39–1.63), 0.73 (0.43–1.23), and 0.88 (0.62–1.26) at 6 months, 1 year, and 2 years post-ESRD transition, respectively, compared with the HD cohort (Figure 4).

### Sensitivity Analyses

A sensitivity analysis was performed to account for change in dialysis modality in the 2 years after ESRD transition. Among the total study population of 2094 patients, 216 (10.3%) patients switched treatment modalities within 2 years of transitioning to ESRD. Among HD, 27 (1.9%) patients changed their treatment modality to PD at a median of 6.8 months after ESRD transition compared with 132 (19.0%) PD patients who switched to HD at a median of 12.2 months. The remaining 57 patient switches were accounted for by transplant (among 33 HD and 24 PD patients).

After adjustment for age, sex, race/ethnicity, preexisting DM and CHF, AKI before transition, eGFR, bicarbonate, and the percent change in eGFR in the 90 days before dialysis, the time-varying analysis found a hazard ratio of 0.94 (0.70–1.24) in mortality within 2 years among PD compared with HD patients (Table 2).

## Conclusion

Our study comparing patients with CKD who transitioned to ESRD in an outpatient setting with an optimal start, found no evidence of a difference in early mortality between PD and HD patients. The clinical information in our study was derived from a real-world clinical care environment among a large racially/ethnically diverse CKD population. In the propensity score–matched cohorts, mortality rates were similar in PD and HD patients at 6 months, 1 year, and 2 years. Using longitudinal information from a single integrated health system, we were able to reliably follow and capture outcomes starting immediately after transition to dialysis without any ascertainment gap. Our findings suggest that dialysis modality is not associated with early ESRD mortality risk among patients with CKD who initiate dialysis with an optimal start on an outpatient basis. Instead, differences in early ESRD transition mortality generally observed may be attributed to predialysis CKD progression along with the timeliness in management and preparation for dialysis to ultimately avoid an urgent start.

Our findings have important implications in that ESRD population is expected to exceed 2 million patients by 2030. Although overall mortality rates among the ESRD population have improved over the past 2 decades, the early period immediately after ESRD continues to represent the most vulnerable and highest mortality risk for patients transitioning to ESRD.^2^ The prevalence of CKD and ESRD continues to rise with overall aging population with multiple comorbidities. However, many patients who progress to ESRD, even with regular nephrology follow-up, do not have a distinct plan at the time of initiating dialysis, resulting in urgent dialysis initiation versus a planned and optimal start.^21^ Thus, optimal start meaning timely preparation with an arteriovenous fistula/graft for HD or PD catheter, should be a priority in advanced CKD management. PD may be an underused modality for renal replacement therapy despite the benefits it offers in terms of lifestyle flexibility, preservation of residual renal function, and even cost savings of more than $20,000 US annually per patient compared with HD.^22^, ^23^, ^24^ However, any mortality benefit over HD probably should not be a topic in the shared decision-making process given the findings to date.

Performing mortality comparisons between PD and HD have been challenging due to inherent biases within the populations that comprise the 2 modalities. The single study that attempted to prospectively randomize HD and PD was prematurely stopped because of low enrollment.^11^ Several observational studies have reported a lower mortality in the first 2 years in PD compared with HD patients.^8^, ^9^, ^10^, ^25^, ^26^ However, after the first 2 years, a similar or even a higher mortality has been observed in PD compared with HD patients.^7^, ^9^, ^27^, ^28^ A Canadian study of more than 10,000 subjects found PD had a survival advantage in the first 2 years compared with HD.^25^ A more recent evaluation of the Canadian registry similarly found PD to have better outcomes compared with HD in the first 2 years, after which PD and HD were found to have similar outcomes.^26^ Jaar *et al.*^8^ found among 1041 participants in the Choices for Healthy Outcomes in Caring for ESRD Patients study found an increased mortality risk in the second year for PD patients compared with HD (2.34 [1.19 – 4.59]) but no difference in the first year. McDonald *et al.*^9^ similarly found no significant increased hazard ratio for mortality between PD and HD in the first year but significantly increased mortality risk for PD versus HD in the second year among their propensity-matched cohort of 16,791 patients. Mehrotra *et al.*^29^ evaluated patients from the US Renal Data System and found no significant difference in risk of death between patients started on PD and HD for up to 5 years of follow-up. However, many of these observations included patients after 90 days of ESRD, thereby introducing an ascertainment bias.^4^ Thus, most prior observations were unable to account for mortality in the critical period immediately after ESRD, which is associated with the highest mortality rates.^5^, ^13^

Differences in findings from mortality comparisons between PD and HD may reflect factors not related to modality type, but rather patient characteristics, differences in predialysis CKD progression and care, and ultimately a selection bias. A recent study conducted among patients who were deemed eligible for either HD or PD demonstrated similar mortality risk between HD and PD patients.^12^ However, the determination of eligibility was based on the judgment of a multidisciplinary team at each dialysis center, thus bias from subjective variations across the centers still existed. In an attempt to account for these differences between PD and HD populations, we included only patients who had an optimal start and initiated dialysis in an outpatient setting. We performed our study among a propensity score–matched population with caliper (<0.001) restriction to minimize the potential bias from a strong treatment-selection process.^30^ It was an attempt to mimic a randomized clinical trial to evaluate an unbiased survival benefit with 80% power to detect 5% difference in mortality within 2 years. In addition, to reflect the everyday clinical practice, a sensitivity analysis was performed in the original population instead of propensity score–matched population, accounting for the effect of dialysis modality switch using an intention-to-treat and a time-varying approach (as treated). Both analyses did not find a higher mortality risk by dialysis modality up to 2 years after ESRD transition. Among a Canadian dialysis population, Quinn *et al*.^31^ found HD and PD were associated with similar survival among 6573 incident dialysis patients who also started dialysis in an outpatient setting. Most PD and HD comparisons could not accurately identify or exclude acutely ill patients who necessitated more urgent ESRD transition.^10^ Less adverse outcomes have been described in patients with urgent start PD compared with those with urgent start HD with a catheter.^21^ Generally, patients who are acutely ill or need dialysis urgently typically start with HD rather than PD.^32^ Thus, the acute-start population may confound mortality comparisons, as the HD subgroups may have been overrepresented with patients who were experiencing more acute or severe circumstances at dialysis. The HD population overall has higher rates of nonoptimal starts (dialysis with central venous catheters) and initiation of dialysis in an inpatient setting.^13^ Catheter dependency remains high particularly during the first 3 months of dialysis initiation.^33^, ^34^ Among our total study population before exclusion, only 17 (2.5%) of the PD patients compared with 41% of HD patients transitioned to ESRD in an inpatient setting.^13^ Couchoud *et al.*^35^ showed that mortality risk increased 50% among elderly patients who had emergent start of dialysis compared with those who had planned dialysis starts. Among these unplanned subpopulations, studies that have evaluated emergent or acute-start PD or HD have found no difference in mortality risk between HD and PD patients.^21^, ^36^, ^37^

Historically, the proportion of patients with ESRD treated with PD has declined. Global PD rates have fallen from as high as 20% in the 1990s to less than 15% in the early 2000s, with the largest decline occurring in developed countries.^38^ However, PD rates have been steadily increasing in the United States for the past 5 years, with PD incidence at 9.6% and prevalence at 7% among the ESRD population.^39^ Among our incident ESRD population in this cohort, 13% of the patients started with PD. One concern with PD is technique failure where up to 10-fold higher technique failure has been described in PD compared with HD.^23^ Up to 25% of patients who initiate PD switch to HD, whereas fewer than 5% of HD patients convert to PD.^8^ Among our cohort, we found 19.0% of PD patients switched to HD compared with 1.9% who switched from HD to PD within 2 years after ESRD. The lower rates of PD as an initial dialysis modality and higher rates of technique failure resulting in conversion to HD have been thought to be due to many factors, including inadequate access to PD facilities, higher infection rates, ultrafiltration failure, and deterioration in patient functional status where they can no longer perform dialysis on their own.^23^ Now that there appears to be a slight rising trend for PD, we must be even more cognizant of patients who have a higher likelihood for these complications. At the same time, PD units should be more proactive toward patient education, volume management, and infection-related preventive strategies.

### Potential Limitations and Strengths

There are several potential limitations to our study that may confound the interpretation of our findings. Despite our attempts to match the PD and HD populations, unmeasured confounders within a real-world clinical environment that led to patient and physician bias toward a dialysis modality cannot be fully accounted for. These include patient characteristics (e.g., education level, socioeconomic status), provider characteristics, not accounting for medication use, and heterogeneity in practice patterns. However, the integrated health system of KPSC does bring about a more standardized care environment among the CKD population. KPSC has an internal CKD registry that identifies patients and gives feedback to providers on management and practice patterns with regard to blood pressure control, anemia management, CKD bone mineral disease management, CKD-related patient education, and preparations for dialysis.^17^, ^40^, ^41^ Given that our study population included incident patients with ESRD who were rather self-selected in that they transitioned to ESRD with an optimal start and as an outpatient, they may not be reflective of the overall ESRD population. Last, we evaluated early ESRD outcomes for only a 2-year window and it would be of interest to determine if there were mortality differences on longer follow-up. We also performed analyses using propensity scoring (inverse probability of treatment weighting) among all incident dialysis patients. Similar results were found for the risk of mortality within 6 months and 2 years. However, a slightly different result was found for mortality risk at 1 year favoring PD (results not shown). Considering the potential bias from extreme weights as the magnitude of treatment selection, we decided to keep the analysis among the propensity score–matched population to minimize the bias where we still had 80% power to detect 5% difference in mortality within 2 years. We also performed conditional logistic regression in addition to our regular logistic regressions model among the propensity score–matched population. Overall, similar crude ORs were obtained (results not shown); however, because of the low event rates at 6 months and 1 year, there was not enough power to do a mortality comparison at 6 months and 1 year. However, the adjusted ORs for 2-year mortality were similar (no difference in mortality ORs) between PD versus HD in the conditional logistic regressions.

Among a propensity-matched CKD population who transitioned to ESRD with an optimal start in an outpatient setting, we found no evidence of a difference in early mortality among PD compared with HD patients. There remained no increased risk in mortality between PD and HD in our time-varying sensitivity analyses that accounted for modality switch over the 2-year observation window. Our findings suggest the importance of predialysis care and the circumstances at ESRD transition over dialysis modality as potential modifiable factors to improve early ESRD outcomes.

## Disclosure

All the authors declared no competing interests.

## Supplementary Material

Table S1 Comparison of propensity score–matched and unmatched patients.

Table S2 Crude mortality rates per month after transition to ESRD by PD, HD, and matched cohorts.
