# Supplementary material for: Early Mortality Among Peritoneal Dialysis and Hemodialysis Patients Who Transitioned With an Optimal Outpatient Start
Source: Kidney Int Rep. 2018 Oct 16;4(2):275–84. doi: 10.1016/j.ekir.2018.10.008 (PMC6365351; doi:10.1016/j.ekir.2018.10.008)
Supplement: Table S1 — Comparison of propensity score–matched and unmatched patients. [file mmc1.docx]

**Supplement Table 1. Comparison of Propensity-Score Matched and Unmatched Patients**

| Characteristics | Unmatched population | Matched Population | *P value* |
| --- | --- | --- | --- |
| N, % | N=1,012 | N=1,082 |  |
| Age, mean (SD) | 63.2 (13.41) | 60.6 (12.34) | <0.001 |
| Female, % | 39.5 | 40.2 | 0.8 |
| Race, % |  |  | 0.3 |
| White | 25.8 | 26.1 |  |
| Black | 20.6 | 20.9 |  |
| Hispanic | 39.8 | 38.2 |  |
| Other | 13.9 | 14.9 |  |
| Diabetes mellitus, % | 78.5 | 77 | 0.4 |
| Hypertension, % | 99.5 | 99.7 | 0.4 |
| Congestive Heart Failure, % | 58 | 53.7 | 0.05 |
| Charlson comorbidity index score, % |  |  | 0.04 |
| 2 | 5.3 | 7.5 |  |
| 3 – 4 | 25 | 27.3 |  |
| ≥ 5 | 69.7 | 65.2 |  |
| Acute kidney injury | 16.5 | 16.2 | 0.8 |
| eGFR, mL/min/1.73m^2^ |  |  | 0.02 |
| < 5 | 5.6 | 4.1 |  |
| 5 – 9 | 55 | 50.6 |  |
| 10 – 14 | 33 | 38.9 |  |
| ≥ 15 | 6.3 | 6.5 |  |
| Potassium, mean (SD) | 4.5 (0.62) | 4.5 (0.61) | 0.7 |
| Ferritin, mean (SD) | 311.6 (286.81) | 311.0 (275.87) | 0.9 |
