# Supplementary material for: Early Mortality Among Peritoneal Dialysis and Hemodialysis Patients Who Transitioned With an Optimal Outpatient Start
Source: Kidney Int Rep. 2018 Oct 16;4(2):275–84. doi: 10.1016/j.ekir.2018.10.008 (PMC6365351; doi:10.1016/j.ekir.2018.10.008)
Supplement: Table S2 — Crude mortality rates per month after transition to ESRD by PD, HD, and matched cohorts. [file mmc2.docx]

**Supplemental Table 2. Crude mortality rates per month after transition to ESRD by PD, HD, and matched cohorts**

| Months after ESRD transition | PD outpatient start | | | HD with AVF/AVG outpatient start | | | Matched PD outpatient start | | | Matched HD with AVF/AVG outpatient start | | |
| --- | --- | --- | --- | --- | --- | --- | --- | --- | --- | --- | --- | --- |
|  | Death | At risk | Deaths/1, 000 persons/year | Death | At risk | Deaths/1, 000 persons/year | Death | At risk | Deaths/1, 000 persons/year | Death | At risk | Deaths/1, 000 persons/year |
| 1 | 3 | 696 | 51.7 | 17 | 1398 | 145.9 | 3 | 541 | 66.5 | 5 | 541 | 110.9 |
| 2 | 1 | 695 | 17.3 | 7 | 1381 | 60.8 | 1 | 538 | 22.3 | 1 | 536 | 22.4 |
| 3 | 3 | 692 | 52.0 | 8 | 1374 | 69.9 | 3 | 537 | 67.0 | 3 | 535 | 67.3 |
| 4 | 2 | 690 | 34.8 | 12 | 1366 | 105.4 | 2 | 534 | 44.9 | 2 | 532 | 45.1 |
| 5 | 3 | 687 | 52.4 | 12 | 1354 | 106.4 | 3 | 532 | 67.7 | 6 | 530 | 135.8 |
| 6 | 2 | 685 | 35.0 | 7 | 1342 | 62.6 | 2 | 529 | 45.4 | 2 | 524 | 45.8 |
| 7 | 2 | 683 | 35.1 | 7 | 1335 | 62.9 | 2 | 527 | 45.5 | 2 | 522 | 46.0 |
| 8 | 5 | 678 | 88.5 | 14 | 1328 | 126.5 | 5 | 525 | 114.3 | 6 | 520 | 138.5 |
| 9 | 0 | 678 | 0.0 | 6 | 1314 | 54.8 | 0 | 520 | 0.0 | 3 | 514 | 70.0 |
| 10 | 2 | 676 | 35.5 | 10 | 1308 | 91.7 | 1 | 520 | 23.1 | 4 | 511 | 93.9 |
| 11 | 2 | 674 | 35.6 | 9 | 1298 | 83.2 | 2 | 519 | 46.2 | 3 | 507 | 71.0 |
| 12 | 1 | 673 | 17.8 | 7 | 1289 | 65.2 | 1 | 517 | 23.2 | 1 | 504 | 23.8 |
| 13 | 5 | 668 | 89.8 | 7 | 1282 | 65.5 | 5 | 516 | 116.3 | 3 | 503 | 71.6 |
| 14 | 0 | 668 | 0.0 | 10 | 1275 | 94.1 | 0 | 511 | 0.0 | 4 | 500 | 96.0 |
| 15 | 4 | 664 | 72.3 | 8 | 1265 | 75.9 | 4 | 511 | 93.9 | 4 | 496 | 96.8 |
| 16 | 5 | 659 | 91.0 | 6 | 1257 | 57.3 | 4 | 507 | 94.7 | 3 | 492 | 73.2 |
| 17 | 5 | 654 | 91.7 | 4 | 1251 | 38.4 | 4 | 503 | 95.4 | 3 | 489 | 73.6 |
| 18 | 6 | 648 | 111.1 | 11 | 1247 | 105.9 | 3 | 499 | 72.1 | 4 | 486 | 98.8 |
| 19 | 3 | 645 | 55.8 | 8 | 1236 | 77.7 | 3 | 496 | 72.6 | 3 | 482 | 74.7 |
| 20 | 7 | 638 | 131.7 | 3 | 1228 | 29.3 | 6 | 493 | 146.0 | 0 | 479 | 0.0 |
| 21 | 1 | 637 | 18.8 | 7 | 1225 | 68.6 | 1 | 487 | 24.6 | 2 | 479 | 50.1 |
| 22 | 3 | 634 | 56.8 | 11 | 1218 | 108.4 | 2 | 486 | 49.4 | 5 | 477 | 125.8 |
| 23 | 5 | 629 | 95.4 | 13 | 1207 | 129.2 | 5 | 484 | 124.0 | 4 | 472 | 101.7 |
| 24 | 6 | 623 | 115.6 | 5 | 1194 | 50.3 | 4 | 479 | 100.2 | 3 | 468 | 76.9 |

AVF- arteriovenous fistula

AVG- arteriovenous graft
